# MonoHER selectively enhances the radiotherapy response in p53 wild-type breast cancer via stabilization of p53

**DOI:** 10.1016/j.ctro.2026.101147

**Published:** 2026-03-15

**Authors:** Chujie Li, Xiaojun Li, Rianne Biemans, Ming Zhang, Ludwig J. Dubois

**Affiliations:** a The M-Lab, Department of Precision Medicine, GROW – Research Institute for Oncology and Reproduction, Maastricht University, Maastricht, the Netherlands; b Department of Pharmacology and Personalized Medicine, NUTRIM – Research Institute of Nutrition and Translational Research in Metabolism, Maastricht University, Maastricht, the Netherlands; c Zhongshan Hospital of Traditional Chinese Medicine, Zhongshan 528400, PR China; d Hainan University-HSF/LWL Collaborative Innovation Laboratory, College of Food Science and Engineering, Hainan University, Haikou 570228, PR China; e Division of Cancer Sciences, School of Medical Sciences, Faculty of Biology, Medicine and Health, The University of Manchester, Manchester M20 4GJ, UK

**Keywords:** monoHER, Breast cancer, Radiotherapy, Radiosensitizer, Apoptosis, DNA damage

## Abstract

**Background:**

Radiotherapy is one of the standard treatments for breast cancer, but its efficacy is limited by tumour radioresistance. Radiosensitizers can improve treatment outcomes. MonoHER, a flavonoid derivative, has shown anticancer potential; however, its role in radiosensitization has not been investigated. Here, we determined the radiosensitizing properties of monoHER in vitro in breast cancer and normal mammary cells.

**Methods:**

Breast cancer cells with different p53 status (MCF7, wild-type; T47D, mutant) and normal mammary cells (MCF10A) were treated with monoHER and radiation. Cell viability, clonogenic survival, apoptosis, and γ-H2AX foci were assessed. Western blotting examined ATM/p53 signalling. Interaction of monoHER with p53 was analysed by molecular docking and CETSA.

**Results:**

MonoHER selectively enhanced radiation-induced cytostatic effects in MCF7 (p < 0.01) cells and in T47D (p < 0.05), but had protective effect in MCF10A (p < 0.01) cells. Combined treatment increased apoptosis (p < 0.001) and DNA damage (p = 0.045) in MCF7 cells, accompanied by upregulation of p-ATM (p = 0.011), p-p53 (p = 0.023), and total p53 (p = 026), while in T47D and MCF10A cells, there is no significant difference. Docking and CETSA confirmed direct binding of monoHER to wild-type p53, increasing its thermal stability. MonoHER alone showed minimal cytotoxicity, suggesting a priming rather than direct killing effect.

**Conclusion:**

MonoHER modestly sensitize only p53-proficient breast cancer cells to radiation by stabilizing and activating p53, highlighting its potential as an adjuvant radiosensitizer.

## Highlights

• MonoHER selectively radiosensitizes p53 wild-type breast cancer cells.
• MonoHER combined with radiation enhances DNA damage, apoptosis, and ATM/p53 pathway activation.
• MonHER directly binds and stabilizes wild-type p53, “priming” cells for irradiation.
• MonoHER has minimal effect on p53-mutant cancer and normal mammary cells.

## Introduction

Globally, breast cancer is the most frequently diagnosed malignancy in women, accounting for nearly one quarter of all female cancer cases, with more than 2 million new cases reported in 2022. Although the therapeutic efficiency has increased, breast cancer remains the leading cause of cancer-related mortality among women, responsible for approximately 15% of cancer deaths worldwide [1]. Despite significant progress in early detection and therapeutic strategies, including surgery, chemotherapy, endocrine therapy, targeted therapy, and radiotherapy, treatment outcomes are still far from optimal, particularly in advanced and resistant cases. Importantly, different molecular subtypes of breast cancer often require distinct therapeutic approaches; however, even with subtype-specific interventions, recurrence and treatment failure remain substantial clinical challenges [2].

Radiotherapy is commonly applied postoperatively to ensure elimination of any remaining breast cancer cell and reduce local recurrence in order to improve survival [3]. Nevertheless, its efficacy is often limited by the intrinsic or acquired radioresistance of tumour cells, as well as by the risk of damage to surrounding normal tissues [4]. To overcome these limitations, combination strategies have been increasingly explored. In particular, the development of radiosensitizers, agents that enhance the sensitivity of tumour cells to radiation without increasing normal tissue toxicity, has attracted considerable attention [5], [6]. Radiosensitizers can act through multiple mechanisms, including amplification of DNA damage, inhibition of DNA repair pathways, or modulation of apoptosis-related signalling [7], [8].

In recent years, flavonoids, a large class of natural polyphenolic compounds, have attracted increasing attention in oncology for their potential to enhance radiosensitivity [9]. Accumulating evidence indicates that several flavonoids, such as hesperidin, can sensitize tumour cells to ionizing radiation by promoting DNA double-strand breaks, suppressing DNA repair, inducing apoptosis, and modulating key signalling pathways, while exhibiting relatively low toxicity to normal tissues [10], [11]. In this study, the flavonoid of interest is monoHER (7-mono-O-(β-hydroxyethyl)-rutoside). MonoHER is a semisynthetic derivative of the flavonoid rutin, a flavonol glycoside, that has a hydroxyethylgroup placed on the 7-oxygen position. Previous research has suggested that monoHER can protect healthy cells against the toxic effects of chemotherapeutics and possess anticancer potential [12]. Here, we aimed to evaluate the radiosensitizing potential of monoHER in breast cancer cells and to explore the molecular mechanisms underlying its activity, with a particular focus on the p53 signalling pathway. We hypothesize that monoHER enhances radiosensitivity in p53 wild-type breast cancer cells by stabilizing and activating p53.

## Materials and methods

### Chemicals and reagents

MonoHER generously provided by Novartis Consumer Health, Nyon, Switzerland, was dissolved in sterile water containing 36 mM NaOH, resulting in a final concentration of 33 mg monoHER/ml with a final pH between 7.8 and 8 [13].

### Cell culture

The human breast cancer cell lines (luminal A) MCF-7 and T-47D were cultured in Dulbecco’s Modified Eagle Medium (DMEM, Sigma-Aldrich) supplemented with 8% fetal bovine serum (FBS, Serana). The human breast epithelial cell line MCF-10A was cultured in DMEM/Ham’s F12 (1:1; Sigma-Aldrich, Gibco), supplemented with 10% FBS, 10 μg/mL insulin (Roche), 20 ng/mL epithelial growth factor (R&D Systems), 0.5 μg/mL hydrocortisone (Sigma-Aldrich) and 100 ng/ml cholera toxin (Sigma-Aldrich). Cell lines were cultured in a humidified incubator at 37°C with 5% CO_2_ and were short tandem repeat-authenticated and confirmed to be mycoplasma-free by using the MycoAlert™ Mycoplasma Detection Kit (Lonza).

Cell viability assay

Cells were seeded in 96-well plates (5000 cells per well), allowed to attach overnight and treated for 24 h with various concentrations of monoHER (12.5–100 µM), or DMEM as vehicle control. 24 h later, cells were irradiated with 6 Gy using a MultiRad225 (225 kV, 17.8 mA, 2.7 Gy/min, 0.3 mm Cu filtration, 15.4 × 15.4 cm FOV, SSD 38 cm; Precision X-Ray Irradiation Inc.) and allowed to grow for another 24 h. Cell viability was determined using the MTT Cell Viability Reagent (Thermofisher). Briefly, media was removed and MTT working solution (diluted in PBS, 1 mg/ml) was added to the wells, and incubated for one hour at 37°C, protected from light. Absorbance (573 nm) was measured using a SpectraMax iD3 microplate reader (Molecular Devices).

### Clonogenic assay

Cells were seeded in 60 mm dishes (5x10^5^ cells/dish), allowed to attach overnight and treated with monoHER (50 µM) for 24 h. Cells were then irradiated with doses ranging from 2 to 6 Gy using a MultiRad225 (225 kV, 17.8 mA, 2.7 Gy/min, 0.3 mm Cu filtration, 15.4 × 15.4 cm FOV, SSD 38 cm; Precision X-Ray Irradiation Inc.). Immediately after irradiation, cells were trypsinized and seeded as single cells in 60 mm dishes in triplicate, at optimized cellular density for clonogenic survival assay. Colonies were allowed to grow for 8–16 days, depending on each cell line. Cells were fixed and stained with 70% ethanol, 0.4% methylene blue, and colonies containing more than 50 cells were counted manually. Surviving fractions were calculated based on plating efficiency and were fit to a linear quadratic model as a function of dose, utilizing weighted least squares regression. The parameters of the clonogenic cell survival curves (A, B) were compared, and significance was tested using a log-rank curve comparison [14].

Apoptosis evaluation by Annexin V/PI

Cell apoptosis was detected using the Annexin V/PI detection kit (Biolegend) according to the manufacturer's instructions. Briefly, 24 h after irradiation, cells were washed twice with cold PBS, suspended in binding buffer, and stained with 5 µL annexin V-FITC and 10 µL PI for 15 min at 25°C in the dark. Annexin V+/PI- cells were considered early apoptotic cells, while Annexin V+/PI+ cells were considered late apoptotic cells. Apoptosis analysis was conducted using a BD FACS Canto Ⅱ flow cytometer.

Immunofluorescence

For γH2AX staining, 24 h after irradiation, cells were fixed in ice-cold methanol for 15 min on ice, followed by permeabilization with 0.2% Tween-20 in PBS for 20 min at RT. Non-specific antibody binding was blocked using 5% normal goat serum and 0.02% Tween-20 in PBS for 30 min at RT. Cells were incubated with a rabbit anti-human γH2AX primary antibody (1:500, Merck, JBW301), followed by detection using goat anti-rabbit Alexa Fluor 488 (1:500, Invitrogen, 11001). Nuclei were counterstained with DAPI (1:500, Merck, D9542).

All samples were imaged using a Leica SPE confocal microscope, and images were processed using Fiji (ImageJ). DNA damage was quantified by counting γH2AX foci per nucleus. Nuclear masks were generated based on DAPI staining, and foci were identified within each nucleus after thresholding.

Molecular docking

Wild-type p53 (PDB ID: 1TUP) was downloaded from the Protein Data Bank, crystallographic waters were removed in PyMOL, and the receptor was prepared in AutoDockTools by adding polar hydrogens, assigning Gasteiger charges, merging non-polar hydrogens, and exporting to PDBQT. The p53 L194F mutant was generated on the AlphaFold Server, aligned to 1TUP, and prepared identically. MonoHER was converted to 3D, protonated for pH 7.4, energy-minimized, and a low-energy conformer was selected; Gasteiger charges were assigned and the ligand was saved as PDBQT. Docking (AutoDock Vina) used a 30 × 30 × 30 Å cubic box centered on Leu194 for the wild type and on Phe194 for the L194F mutant. Poses were ranked by Vina score, and chemically reasonable binding modes without steric clashes and with plausible interactions around residue 194 were retained for visualization and analysis. Results are reported as the predicted binding force (Vina binding affinity, kcal mol⁻^1^).

Cellular thermal shift assay (CETSA)

Cells were seeded in 60 mm dishes (1x10^6^ cells/dish) and treated with an FBS-free medium containing DMEM or 50 μM monoHER for 2 h. After treatment, the cells were collected, washed, and resuspended in 300 μL of PBS. Each 30 μL suspension was aliquoted into 0.2 mL PCR tubes and heated in a PCR machine at the indicated temperature (43–70 ℃) for 5 min. 5 μL of RIPA buffer (50 mM HEPES-KOH, pH 7.5, 150 mM KCL, 1 Mm EDTA, 2 mM mercaptoethanol, 0.2% Tween-29 (Sigma Aldrich, R0278) with protease inhibitor (Roche, 4693124001) was added to each tube, and the precipitated proteins were removed by centrifugation at 15000g for 15 min at 4 ℃ after thorough mixing. Subsequent supernatant manipulation was based on general western blot experiments [15].

Immunoblotting

24 h after irradiation, cells were lysed in RIPA buffer and sonicated 3 × 5 s at 10 MHz. Samples (30 µg) in Laemmli sample buffer were loaded on a polyacrylamide gel, separated by SDS-PAGE, transferred to PVDF membranes (VWR, 10600023) using a wet transfer system. Membranes were blocked in 5% non-fat milk in TBST for 1 h at RT and incubated overnight at 4°C with primary antibodies against p-ATM (1:2000; Cell Signaling Technology, 5883), p-p53 (1:1000; Cell Signaling Technology, 9284) and p53 (1:1000; Cell Signaling Technology, 9282). α-Tubulin (1:1000, Sigma Aldrich, T7451) or Vinculin (1:1000, Sigma Aldrich, V9131) were used as loading controls. Primary antibodies were visualized using HRP-link secondary antibodies (anti-rabbit, anti-mouse, 1:5000; Cell Signaling Technology, 7076S, 7074S). Super Signal West Pico chemiluminescent substrate (Thermo Scientific, 34579) was used for detection [12], [16].

Statistical analyses

Statistical analysis was performed using GraphPad Prism software (version 9). All results are expressed as mean ± SEM. The results were evaluated using one-way ANOVA with Tukey’s multiple comparison test or Student's *t*-test when appropriate. Values of p < 0.05 were considered statistically significant.

## Results

### MonoHER selectively sensitizes breast cancer cells to radiation

**Fig. 1:**
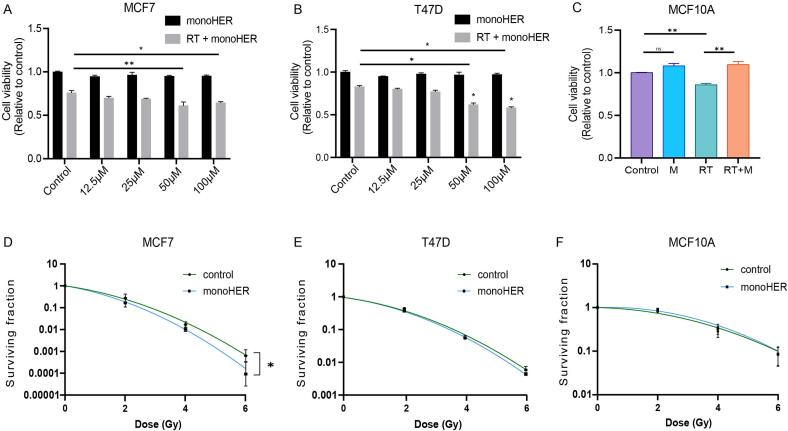
**Monoher selectively enhances radiation-induced cytotoxicity in breast cancer cells.** (A-B) Cell viability of MCF7 and T47D cells treated with increasing concentration of monoHER (12.5–100 μM) alone or in combination with 6 Gy radiation (RT). (C) Cell viability of MCF10A cells treated with 50 μM monoHER (M), 6 Gy (RT) or combination of monoHER and 6 Gy (RT + M). (D–F) Clonogenic surviving fraction of irradiated MCF7, T47D, and MCF10A cells with or without monoHER pretreatment. Data are presented as mean ± SEM from three independent experiments. *p < 0.05, **p < 0.01.

To evaluate the selectivity of monoHER as a radiosensitizer, MCF7, T47D and non-tumorigenic MCF10A cells were treated with monoHER alone or in combination with 6 Gy radiation (RT). In MCF7 and T47D cells, monoHER (12.5–100 µM) alone did not affect cell viability. However, at 50 and 100 µM MonoHER combined RT significantly reduced cell viability in both cancer cell lines (Fig. 1A and 1B) in a concentration-dependent manner. In contrast, 50 µM MonoHER protected non-tumorigenic MCF10A cells against the reduction in cell viability by RT (Fig. 1C).

To further evaluate radiosensitization, clonogenic assays were performed. In MCF7 cells, monoHER significantly decreased the surviving fraction after RT (p < 0.05) (Fig. 1D), indicating enhanced radiosensitivity. In contrast, no significant effect of monoHER was observed in T47D (p = 0.051; Fig. 1E) or MCF10A cells (p = 0.833; Fig. 1F). Together, these results indicate that monoHER selectively enhances radiation-induced cytotoxicity in breast cancer cells, particularly in p53 wild-type MCF7 cells, while sparing normal epithelial cells.

### Combined monoHER and radiation treatment enhances apoptosis more in p53 wild-type cancer cells

**Fig. 2:**
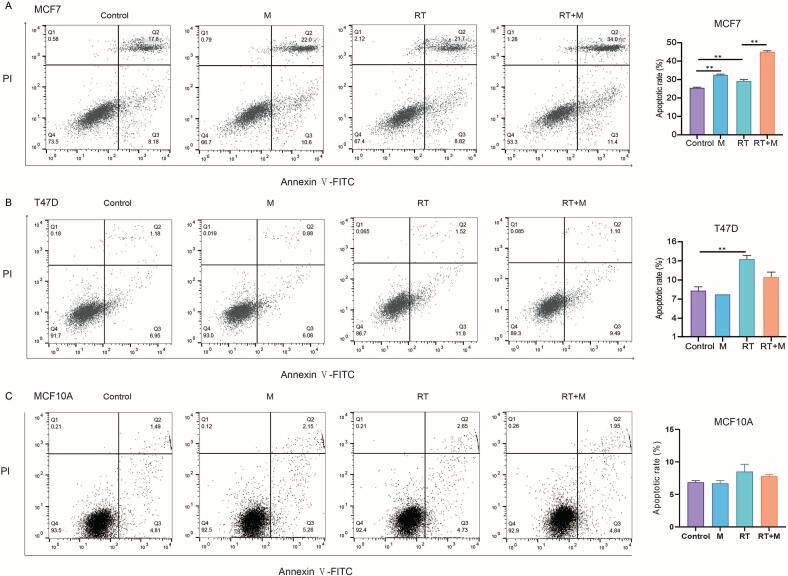
**Effect of monoHER combined with radiation on apoptosis in breast cancer and normal cells.** Annexin V/PI assays detected at 24 h post-irradiation in MCF7 (A), T47D (B) and MCF10A (C) cells treated with monoHER (M) alone or 6 Gy (RT) or combination of monoHER and 6 Gy (RT + M). Data are presented as mean ± SEM from three independent experiments. **p < 0.01.

**Fig. 3:**
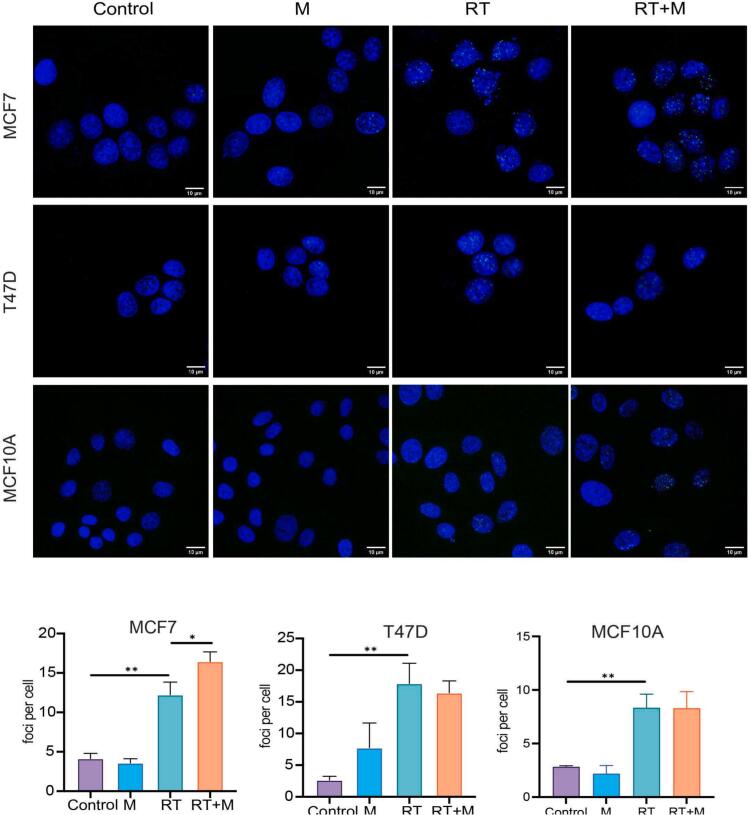
**Effect of monoHER (M) combined with radiation (RT) on γ-H2AX immunofluorescence staining** (at 24 h post-irradiation time point) **in breast cancer and normal cells.** Data are presented as mean ± SEM from three independent experiments (20 cells/experiment). *p < 0.05, **p < 0.01.

Apoptosis induction by monoHER and radiation was assessed using Annexin V/PI staining. In MCF7 cells, treatment with monoHER (p < 0.001) or RT (p < 0.001) alone increased apoptosis compared with vehicle control (Fig. 2A). The combination further increased apoptosis to ∼ 40% (p < 0.001). In T47D cells, RT alone increased apoptosis compared with the control (p = 0.002), but the combination treatment did not further increase the apoptotic rate (Fig. 2B). In MCF10A cells, no differences were observed between the experimental groups (Fig. 3B). These results indicate that the p53 wild-type MCF7 cells are more sensitive to the pro-apoptotic effect of the combined treatment compared the p53 mutant T47D cells and MCF10A cells.

### MonoHER enhanced radiation-induced DNA damage in p53 wild-type cancer cells

DNA damage was evaluated by γ-H2AX immunofluorescence staining. RT markedly increased γ-H2AX foci formation in MCF7 (p < 0.001), T47D (p < 0.001) cancer cells and MCF10A cells (p = 0.029) as compared to the vehicle controls (Fig. 3). In MCF7 cells, the combination of monoHER and radiation further increased γ-H2AX foci (p = 0.045). In contrast, monoHER had no effect on radiation-induced foci formation in T47D cells (p = 0.978) or MCF10A cells (p > 0.999). These findings indicate that monoHER enhances radiation-induced DNA damage selectively in p53 wild-type MCF7 cells.

### monoHER regulated radiation-induced activation of the ATM/p53 pathway in p53 wild-type cancer cells

**Fig. 4:**
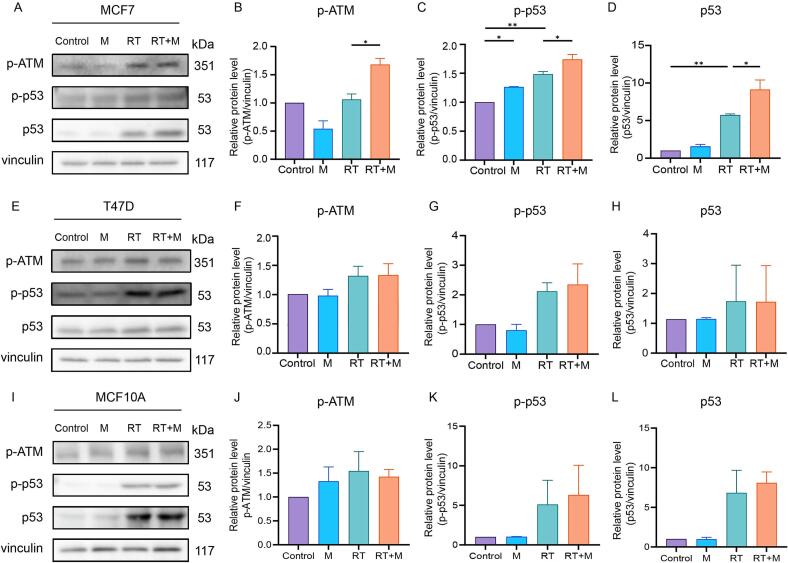
**Different protein expression of p-ATM, p-**p**53 and p53 in breast cancer cells treated with combination of monoHER (M) and radiation (RT).** Representative Western blots of p-ATM, p-p53 and p53 in MCF7 (A), T47D (E) and MCF10A (H) cells. Quantification of p-ATM (B), p-p53 (C) and p53 (D) protein expression in MCF7 cells. Quantification of p-ATM (E), p-p53 (F) and p53 (G) protein expression in T47D cells. Quantification of p-ATM (I), p-p53 (J) and p53 (K) protein expression in MCF10A cells. Data are presented as mean ± SEM from three independent experiments. *p < 0.05, **p < 0.01.

To investigate the molecular mechanism underlying the enhanced radiosensitivity by monoHER, the expression of phosphorylated ATM (p-ATM), phosphorylated p53 (p-p53) and total p53 were evaluated by Western blotting. As shown in Fig. 4A, radiation alone increased the levels of p-p53 (p < 0.001) and p53 (p = 0.004) in MCF7 cells. Importantly, combined treatment with monoHER and radiation significantly increased both p-ATM (p = 0.011) and p-p53 (p = 0.023) compared to radiation alone. In addition, the total p53 protein level was also elevated by radiation and further increased (p = 0.026) upon combined treatment (Fig. 4A-4D), indicating activation and accumulation of p53 in response to combined treatment. In T47D and MCF10A cells, although radiation induced a modest increase in p-ATM and p-p53 levels, monoHER did not further enhance these changes (Fig. 4E–L). Similarly, total p53 levels remained unchanged after the combined treatment in these two cell lines. These results suggest that monoHER enhances radiation-induced activation and accumulation of p53 either through ATM signaling or stabilizing p53 in p53 wild-type MCF7 cells but has little effect in p53 mutant T47D cancer cells and p53 wild-type MCF10A normal cells.

### MonoHER binds preferentially to wild-type p53 and promotes cell death via stabilizing it

**Fig. 5:**
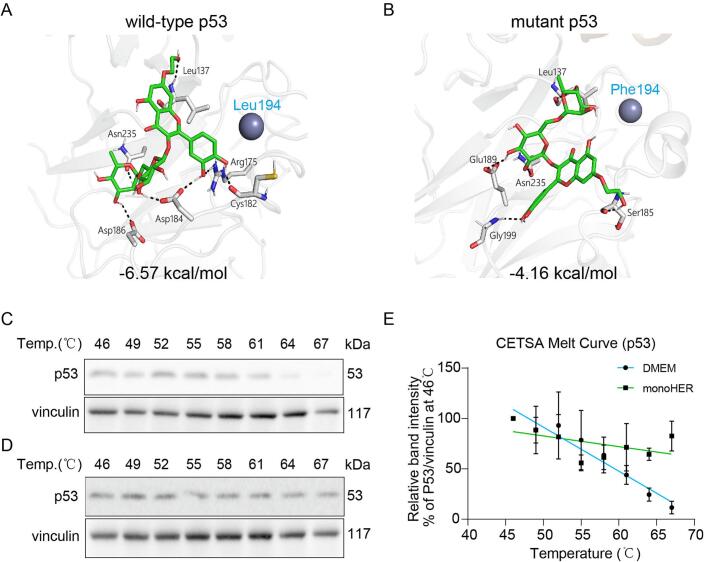
**Monoher interacts with p53.** Biomolecular interactions of monoHER with wild-type p53 (A) and mutant p53 (B). Docking scores are indicated at the bottom of the image. Representative Western blots and quantification (E) of the CETSA experiment with cell lysates of MCF7 cells, which were treated with DMEM (C) or monoHER (D). Data are presented as mean ± SEM from three independent experiments.

To investigate if monoHER interacts differently with wild-type and mutant p53, molecular docking was performed to compare the interaction of monoHER with wild-type and mutant p53 proteins. MonoHER strongly binds to the wild-type p53 core domain (docking score of –6.57 kcal/mol), forming multiple hydrogen bonds with key residues such as Leu 137, Asn 235, Arg175, Asp184, Asp186 and Cys182 (Fig. 5A). In contrast, docking to the mutant p53 protein yielded a lower binding score (–4.16 kcal/mol), and fewer interactions, mainly with Leu137, Ser185, Glu198, Gly199 and Asn235, suggesting a reduced affinity.

CETSA confirmed this interaction in MCF7 cells: monoHER treatment (slope −0.01) increased the stability of p53 proteins in MCF7 cells compared to vehicle control (slope −0.04) (Fig. 5C-E).

Together, these findings indicate that monoHER preferentially binds to and stabilizes wild-type p53, which may contribute to its radiosensitizing effect in p53-proficient breast cancer cells.

## Discussion

In this study, we investigated the radiosensitizing modulatory effect of monoHER and its underlying mechanism in breast cancer cells with different p53 status. Our results demonstrate that monoHER selectively modulates the RT response in p53 wild-type MCF7 cancer cells, while having little or no impact on p53 mutant T47D cancer cells. Specifically, the combination of monoHER and RT significantly reduced cell viability and clonogenic survival at higher radiation dose in MCF7 cells, suggesting a true modest radiosensitizing effect. In contrast, although a slight reduction in cell viability was observed in T47D cells at high concentrations of monoHER, clonogenic survival remained unaffected. This discrepancy could be explained by the distinct biological endpoints. The cell viability (MTT) assay reflects short-term metabolic activity, whereas the clonogenic assay assesses long-term proliferative capacity and is considered the gold standard for evaluating radiosensitivity. The observed effect in T47D cells is likely cytostatic or metabolic rather than cytotoxic. This also was further confirmed by the apoptosis results in T47D cells, which show limited induction of apoptotic cell death under the same conditions. Mechanistically, monoHER increased RT-induced apoptosis and DNA damage in MCF7 cells, effects that were absent in T47D cells, highlighting a key role for p53 in mediating the cellular response to monoHER. Importantly, monoHER did not affect colony formation, irradiation-induced γH2AX foci formation and apoptosis of p53 wild-type normal MCF10A breast normal epithelial cells, likely due to their intact DNA repair capacity and distinct p53 functional outputs, which favour cell cycle arrest and repair rather than apoptosis [17], [18]. This suggests that the radiosensitizing effect of monoHER is not solely determined by p53 status, but also by the cellular context in which p53 operates.

The tumour suppressor p53 is at the hub of cellular signalling networks that are activated by stress signals including DNA damage [19]. In unstressed cells, p53 is kept at low levels by its negative regulator Mdm2 [20]. Upon DNA damage, p53 is stabilized and activated to function primarily as a transcription factor, regulating expression of downstream target genes. This leads to different cellular outcomes such as cell cycle arrest and apoptosis [21]. Cell cycle arrest facilitates DNA repair and promotes survival, whereas apoptosis efficiently removes irreparably damaged cells. Under stress conditions, p53 activation occurs through upstream mediators, many of which induce posttranslational modification that disrupts the p53-Mdm2 interaction. For example, DNA double-strand breaks (DSBs) trigger rapid autophosphorylation and activation of ataxia-telangiectasia mutated protein (ATM) [22], which then phosphorylates p53 [23], [24] and other substrates. These events lead to dissociation of the p53-Mdm2 complex, stabilizing p53 and increasing its protein levels [25]. In addition to such upstream signalling, monoHER may directly stabilize p53 by binding to it, altering its conformation to enhance thermal stability and prevent degradation, similar to compounds, like RITA [26].

Western blot analysis confirmed that the combination of monoHER and radiation significantly increased phosphorylation of ATM and p53, as well as total p53 protein levels in MCF7 cells. These data suggest that monoHER might enhance DNA damage signalling through the ATM/p53 pathway, contributing to cell death. In contrast, monoHER did not further increase p-ATM or p-p53 levels in T47D cells, indicating that the lack of radiosensitization in these cells may attributed to the presence of mutant p53. Although the levels of p-p53 in T47D cells increase more than in MCF7 cells, mutant p53 often shows elevated basal or radiation-induced stability and phosphorylation of p53 that do not translate into functional transcriptional or apoptotic activity. Under these conditions, a ceiling effect may limit further increases in p-p53, and monoHER does not further modulate the ATM–p53 signalling axis beyond irradiation alone. Furthermore, monoHER did not alter irradiation-induced protein expression levels of p-ATM, p-p53, and total p53 compared to irradiation alone in MCF10A cells, indicating that monoHER does not amplify the DNA damage response or activate downstream p53 signalling in normal epithelial cells, despite their wild-type p53 status.

To explore whether monoHER directly interacts with wild-type p53, we performed molecular docking and CETSA analyses. Docking results revealed a stronger binding affinity of monoHER to the wild-type p53 compared with the mutant form, and CETSA confirmed that monoHER increased the thermal stability of p53 in MCF7 cells. Although monoHER was shown to bind and stabilize wild-type p53, treatment with monoHER alone did not markedly reduce cell viability or clonogenic survival. This apparent discrepancy may be explained by the fact that p53 activation alone is not sufficient to induce substantial cytotoxicity in the absence of DNA damage. MonoHER may stabilize p53 and maintain it in a conformation that is more responsive to stress, but a strong upstream signal such as radiation-induced DNA damage is required to fully activate the p53 pathway. In other words, monoHER “primes” the cells by facilitating p53 stabilization, thereby enabling a stronger p53 response once DNA damage is introduced by irradiation. Similar priming effects have been reported for other radiosensitizers that modulate p53 without inducing significant cytotoxicity by themselves [27]. These findings suggest that monoHER directly interacts with and stabilizes wild-type p53, thereby enhancing its DNA damage dependent activation. Consequently, monoHER may prolong p53 activation following irradiation and amplify downstream apoptotic signalling. Overall, our results suggest that monoHER can stabilize p53 and prevent its degradation, but only in p53 wild-type cancer cells.

Several limitations of this study should be acknowledged. First, all experiments were performed *in vitro* using breast cancer cell lines and immortalized mammary epithelial cells. Therefore, the radiosensitizing effects of monoHER need to be validated *in vivo* to better capture the complexity of tumour microenvironments. Second, we only investigated two breast cancer cell lines with different p53 status, which may not fully capture the heterogeneity of breast cancer. Additional cell models with different genetic backgrounds should be included to strengthen the generalizability of our findings. Third, although molecular docking and CETSA support a direct interaction between monoHER and p53, the precise binding site and structural consequences can only be confirmed by biophysical or crystallographic analyses.

## Conclusion

This study demonstrates that monoHER selectively modulates the radiosensitivity of breast cancer cells in a p53-dependent manner. MonoHER amplified radiation-induced DNA damage, activated the ATM/p53 pathway, and promoted apoptosis in MCF7 cells with wild-type p53, whereas no comparable effects were observed in T47D (mutant p53) or normal MCF10A cells. These findings provide mechanistic insight into a context-dependent radiosensitizing effect of monoHER and support its potential application in combination with radiotherapy for the treatment of p53 wild-type breast cancer.

## CRediT authorship contribution statement

**Chujie Li:** Conceptualization, Methodology, Software, Validation, Formal analysis, Writing – original draft, Writing – review & editing. **Xiaojun Li:** Conceptualization, Methodology, Software, Validation, Formal analysis, Writing – original draft. **Rianne Biemans:** Methodology. **Ming Zhang:** Writing – review & editing, Supervision. **Ludwig J. Dubois:** Conceptualization, Validation, Writing – review & editing, Supervision.

## Funding

This work was supported by 10.13039/501100001809National Natural Science Foundation of China (32201977). Chujie Li was supported by the 10.13039/501100004543China Scholarship Council (No. 202108440109).

## Declaration of competing interest

The authors declare the following financial interests/personal relationships which may be considered as potential competing interests: [LJD has, outside of the submitted work, shares in the companies Convert Pharmaceuticals and LivingMed Biotech, and he is co-inventor of a non-issued patent on LSRT (N2024889)].
